# Early onset of immune-mediated diseases in minority ethnic groups in the UK

**DOI:** 10.1186/s12916-022-02544-5

**Published:** 2022-10-13

**Authors:** Archana Sharma-Oates, Dawit T. Zemedikun, Kanta Kumar, John A. Reynolds, Avinash Jain, Karim Raza, John A. Williams, Laura Bravo, Victor Roth Cardoso, Georgios Gkoutos, Krishnarajah Nirantharakumar, Janet M. Lord

**Affiliations:** 1 Institute of Inflammation and Ageing, University of Birmingham, Birmingham, B15 2TT UK; 2 School of Biosciences, University of Birmingham, Birmingham, B15 2TT UK; 3 Institute of Applied Health Research, University of Birmingham, Birmingham, B15 2TT UK; 4 Institute of Clinical Sciences, University of Birmingham, Birmingham, B15 2TT UK; 5 Department of Rheumatology, Sandwell and West Birmingham NHS Trust, Birmingham, UK; 6 SMS Medical College, Jaipur, Rajasthan India; 7 MRC-Versus Arthritis Centre for Musculoskeletal Ageing Research, University of Birmingham, Birmingham, B15 2TT UK; 8 NIHR Birmingham Biomedical Research Centre, University Hospital Birmingham and University of Birmingham, Birmingham, UK; 9 Health Data Research UK, Midlands Site, Birmingham, B15 2TT UK; 10 Institute of Cancer and Genomic Sciences, University of Birmingham, Birmingham, B15 2TT UK

**Keywords:** Autoimmune inflammatory diseases, Immune-mediated diseases, Ethnicity, South Asian, African-Caribbean, Diagnosis, Rheumatic diseases, Ageing

## Abstract

**Background:**

The prevalence of some immune-mediated diseases (IMDs) shows distinct differences between populations of different ethnicities. The aim of this study was to determine if the age at diagnosis of common IMDs also differed between different ethnic groups in the UK, suggestive of distinct influences of ethnicity on disease pathogenesis.

**Methods:**

This was a population-based retrospective primary care study. Linear regression provided unadjusted and adjusted estimates of age at diagnosis for common IMDs within the following ethnic groups: White, South Asian, African-Caribbean and Mixed-race/Other. Potential disease risk confounders in the association between ethnicity and diagnosis age including sex, smoking, body mass index and social deprivation (Townsend quintiles) were adjusted for. The analysis was replicated using data from UK Biobank (UKB).

**Results:**

After adjusting for risk confounders, we observed that individuals from South Asian, African-Caribbean and Mixed-race/Other ethnicities were diagnosed with IMDs at a significantly younger age than their White counterparts for almost all IMDs. The difference in the diagnosis age (ranging from 2 to 30 years earlier) varied for each disease and by ethnicity. For example, rheumatoid arthritis was diagnosed at age 49, 48 and 47 years in individuals of African-Caribbean, South Asian and Mixed-race/Other ethnicities respectively, compared to 56 years in White ethnicities. The earlier diagnosis of most IMDs observed was validated in UKB although with a smaller effect size.

**Conclusion:**

Individuals from non-White ethnic groups in the UK had an earlier age at diagnosis for several IMDs than White adults.

**Supplementary Information:**

The online version contains supplementary material available at 10.1186/s12916-022-02544-5.

## Background

The prevalence of immune mediated diseases (IMDs) is increasing worldwide together with associated mortality and morbidity rates. IMDs are a group of diseases that cause damage to tissues and organs in response to self-antigens [1]. Studies into individual diseases such as systemic lupus erythematosus (SLE) and rheumatoid arthritis (RA) have shown that their incidence and prevalence differ between ethnic groups [2, 3]. Recent studies report the highest incidence of SLE to be in the African-Caribbean population [4, 5] and the highest incidence of RA in the South Asian population [3]. Other studies have reported the incidence of IMDs such as vitiligo and autoimmune thyroid disease (AIT) to be higher in these ethnic groups [3, 6].

Importantly, several studies indicate that severe disease involving major organs occurs at a younger age in different ethnic populations, which may indicate an earlier age of onset [7–10]. If the onset of IMDs is earlier in certain ethnic groups, this would likely result in longer disease duration and increase the risk of long-term disease complications which has implications for healthcare utilisation. Additionally, if individuals from these ethnic groups develop IMDs at an earlier age, this may suggest that ethnicity influences immune responses relevant to disease pathogenesis as well as potentially more broadly. However, we are not aware of any large-scale epidemiological studies that assess differences in diagnosis ages between different ethnic groups.

We therefore initiated a study to compare diagnosis ages of IMDs between different ethnic groups within the UK.

## Methods

Our study applied data from IQVIA Medical Research Data (IMRD-UK) [Scientific Review Committee Reference Number: 18THIN064], which is an electronic health records (EHR) database of primary care patients in the UK. IMRD-UK is representative of the UK population in terms of demographic structure and common morbidity prevalence [11]. The ethnicity of patients was recorded by general practices through self-reporting by patients. Information relating to symptoms, diagnoses and referrals are recorded within IMRD-UK using Read Codes, a clinical hierarchy coding system [12].

This study was a population-based retrospective study with a cohort of ~4.5 million. The study period was set between 1st January 2006 (ethnicity recording at optimal completeness) and 31st December 2020, and patients of all ages were considered for the study. General practices in this study were included 12 months after their instalment of EHR or 12 months from the practice’s acceptable mortality recording dates to reduce under-recording of events [13, 14]. In addition, patients were only allowed to enter the study after 12 months of registration with an eligible general practitioner. Furthermore, a patient with more than one autoimmune condition could contribute to more than one disease group.

Ethnicity in this study was categorised broadly into the four most common groups based on the 2011 UK census classification [15]: (1) White (British, Irish, other White); (2) South Asian (Bangladeshi, Pakistani, Indian, Sri Lankan, British Asian or other South Asian); (3) African-Caribbean (Black African, Black Caribbean, Black British or other Black people); and (4) Mixed-race/Other ethnic groups (including Chinese, Vietnamese, and other South-East Asian). The most recent record of ethnicity was utilised in this study.

Outcomes of IMDs, as well as fibromyalgia (FM), as a comparator chronic but non-autoimmune disease, were identified by relevant Read Codes (Additional file 1). IMDs included in the analysis were: AIT; coeliac disease; inflammatory bowel disease (IBD); myasthenia gravis (MG); multiple sclerosis (MS); psoriasis; pernicious anaemia (PA); RA; SLE; Sjogren’s syndrome; and vitiligo. Read Code lists for all IMDs considered are detailed in a previous study [16]. Patients were followed up from index date (study entry) until the earliest of the following end points which marked the exit date: diagnosis date, death date, study end date, date patient transferred from GP, or last date of data collection from a given GP.

UK Biobank (UKB) [application number: 31224] is a cross-sectional study of approximately 500,000 participants, aged between 40 and 69 years, that were recruited between 2006 and 2010 [17]. The data collected include detailed information on health and lifestyle as well as genetic data.

The dates of the First Occurrence of Health Outcomes data were extracted using the ICD-10 codes from category 1712 (Table S1). Ethnic background was determined from field 21000. The data sources included ICD-10 codes obtained from hospital inpatient data, death Register records, self-reported and from read codes in the primary care data.

### Statistical analysis

Baseline covariates were summarised using appropriate descriptive statistics, stratified by ethnicity. Median (interquartile range, IQR) for continuous variables due to their skewed distribution and frequency (%) for categorical variables were used to describe the baseline characteristics. Linear regression was used to provide unadjusted and adjusted estimates of age at diagnosis by ethnicity. Checks were performed to ensure that the assumptions of linear regression were not violated, and no evidence of heteroscedasticity was found. Additional tests confirmed that the residuals (errors) of the regression line were approximately normally distributed. Baseline record of sex, smoking, body mass index (BMI) and social deprivation (Townsend quintiles) were considered as potential confounders in the association between ethnicity and age at diagnosis of the outcome conditions. Patients in which the date of diagnosis preceded the baseline date were excluded from the analysis. In addition, missing data from each covariate were treated as a separate missing category and included in the regression analysis to enable the same number of patients to be compared across the different analyses. All analyses were performed using Stata 16 SE, and a *p*-value <0.05 was considered statistically significant.

Comparisons of the ages at IMDs diagnosis were between the three main groups: South Asian, African-Caribbean, and Mixed-race/Other and the White ethnic group, both with and without adjustment for relevant potential confounding variables.

## Results

### Study cohort characteristics

The baseline study characteristics include the highest proportion of adults aged over 50 years in White (18.5%) group followed by South Asian (7.7%), African-Caribbean (6.8%) and Mixed-race/Other (5.5%) groups (Table 1). The African-Caribbean group contain the highest proportion (13.8%) of obese participants (BMI ≥30) followed by White (12.3%), South Asian (7.8%) and Mixed-race/Other (6.3%) groups. However, BMI data were missing or considered implausible (BMI values < 14 or > 75) from a considerable proportion of the cohort (Table 1). The proportion of participants who were most socially deprived was higher amongst the three non-White ethnic groups in comparison to White group although information was missing for a considerable proportion of participants (Table 1). The highest proportion of smokers was from the White group and the highest proportion of non-smokers were from the South Asian group (Table 1).

**Table 1:** IMRD-UK study characteristics of the age at diagnoses of IMDs in different ethnic groups

Description	White	Mixed-race/Other	African-Caribbean	South Asian
Total patients (*n*)	(*n*=3,902,748)	(*n*=199,520)	(*n*=172,329)	(*n*=250,755)
Sex *n* (%)				
Male	1,886,048 (48.33)	92,835 (46.53)	82,722 (48.00)	131,192 (52.32)
Female	2,016,700 (51.67)	106,685 (53.47)	89,607 (52.00)	119,563 (47.68)
Age (years) median (IQR)	32.00 (19.00–49.00)	27.00 (14.00–37.00)	29.00 (13.00–40.00)	29.00 (14.00–38.00)
Age category (years) *n* (%)				
Under 18	937,782 (24.03)	57,049 (28.59)	52,651 (30.55)	68,599 (27.36)
18–34	1,170,835 (30.00)	82,026 (41.11)	56,095 (32.55)	100,714 (40.16)
35–54	1,074,047 (27.52)	49,430 (24.77)	51,912 (30.12)	62,251 (24.83)
55 and above	720,084 (18.45)	11,015 (5.52)	11,671 (6.77)	19,191 (7.65)
BMI (kg/m^2^) median (IQR)	25.00 (22.00–28.00)	23.00 (20.00–26.00)	26.00 (23.00–29.00)	24.00 (21.00–27.00)
BMI Category (kg/m^2^) *n* (%)				
Underweight/normal (up to 24.9)	1,150,095 (29.47)	74,866 (37.52)	40,217 (23.34)	81,302 (32.42)
Overweight (25–29.9)	771,355 (19.76)	29,475 (14.77)	33,740 (19.58)	48,327 (19.27)
Obese (30 and above)	478,606 (12.26)	12,638 (6.33)	23,698 (13.75)	19,617 (7.82)
Missing or implausible	1,502,692 (38.50)	82,541 (41.37)	74,674 (43.33)	101,509 (40.48)
Townsend quintiles *n* (%)				
1 (least deprived)	634,187 (16.25)	16,278 (8.16)	7390 (4.29)	20,472 (8.16)
2	610,020 (15.63)	17,024 (8.53)	9793 (5.68)	22,395 (8.93)
3	660,340 (16.92)	28,424 (14.25)	22,160 (12.86)	39,543 (15.77)
4	618,324 (15.84)	35,568 (17.83)	33,686 (19.55)	51,656 (20.60)
5 (most deprived)	466,411 (11.95)	33,452 (16.77)	43,131 (25.03)	44,886 (17.90)
Missing	913,466 (23.41)	68,774 (34.47)	56,169 (32.59)	71,803 (28.63)
Smoking status *n* (%)				
Non-smoker	1,643,268 (42.11)	107,008 (53.63)	98,327 (57.06)	149,154 (59.48)
Smoker	746,446 (19.13)	24,719 (12.39)	16,499 (9.57)	23,555 (9.39)
Ex-smoker	561,268 (14.38)	14,381 (7.21)	10,051 (5.83)	12,335 (4.92)
Missing	951,766 (24.39)	53,412 (26.77)	47,452 (27.54)	65,711 (26.21)

### Age at diagnosis by ethnicity

**Fig. 1:**
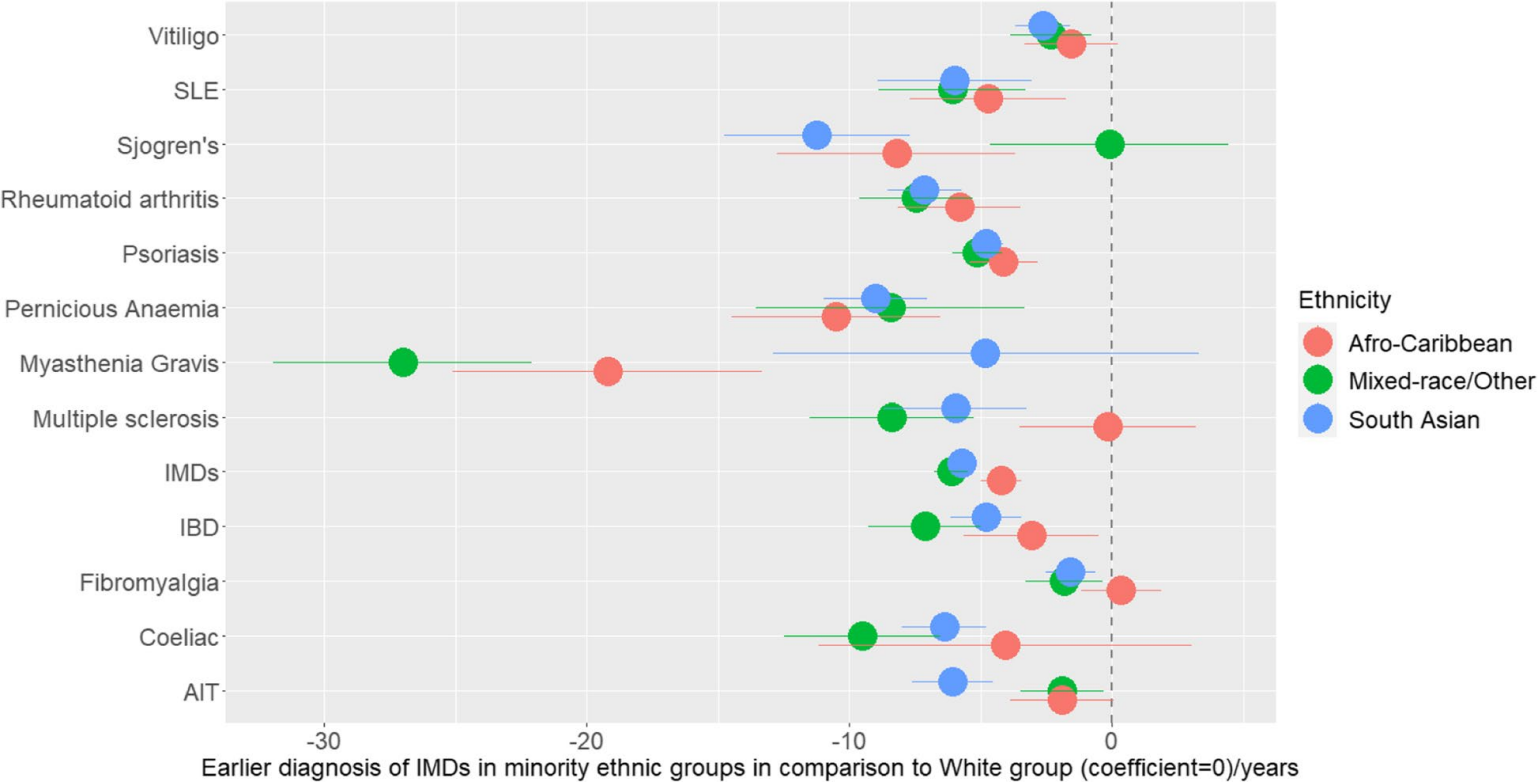
Coefficient plot demonstrating the earlier diagnoses age of IMDs (*Y*-axis) in the different ethnic minority groups with comparison to the reference White group (indicated by dashed line to the right). The figure was generated using the age at diagnosis after adjustments for confounding variables: BMI, smoking status, sex and social deprivation

There were significant differences in the ages at diagnosis of IMDs between the four ethnic groups after adjusting for sex and potential lifestyle confounders (smoking, BMI and levels of social deprivation) (Table 2). Age at disease diagnosis for all IMD cases combined, adjusted for risk factors, revealed that patients were diagnosed on average 4 years earlier in the African-Caribbean group and 6 years earlier in both the South Asian and Mixed-race/Other groups than those from the White group (Table 2 and Fig. 1). Age at diagnosis of almost all IMDs was earlier in patients of South Asian ethnicity (Fig. 1). The Mixed-race/Other group followed a similar trend to South Asians except for Sjogren's syndrome where the diagnosis age was not statistically different to the White group. Although the African-Caribbean group also followed a similar trend to the other two non-White groups, they differed for MS and AIT where the diagnosis age was not significantly different to the White group (Table 2). In contrast, there was no difference between the White and African-Caribbean groups in the diagnosis age for FM. The difference in FM diagnosis age was small at 1.6 years and 1.8 years in the South Asian, Mixed-race/Other groups, respectively, in comparison to the White group (Table 2, Fig. 1).

**Table 2:** Unadjusted and adjusted differences in age at disease diagnoses in different ethnic groups using White ethnicity as the reference group in the IMRD-UK cohort

	***N*** (%)	Age/years Mean (SD)	Unadjusted models				Adjusted models^a^			
			Coefficient	***P***-val.	(95% CI)		Coefficient	***P***-val.	(95% Cl)	
**Systemic lupus erythematosus**										
White	1213 (81.7)	48.5 (16.2)	Ref.				Ref.			
Mixed-race/Other	78 (5.3)	41.4 (11.9)	− 7.00	<0.001	− 9.78	− 4.22	− 6.08	<0.001	− 8.87	− 3.28
African-Caribbean	95 (6.4)	42.6 (15.8)	− 5.81	<0.001	− 9.11	− 2.51	− 4.72	<0.001	− 7.69	− 1.76
South Asian	99 (6.7)	40.1 (15.7)	− 8.35	<0.001	− 11.56	− 5.14	− 6.00	<0.001	− 8.93	− 3.06
**Vitiligo**										
White	3563 (71.1)	38.4 (19.5)	Ref.				Ref.			
Mixed-race/Other	303 (6.1)	30.3 (19.9)	− 8.13	<0.001	− 10.46	− 5.79	− 2.34	<0.001	− 3.88	− 0.80
African-Caribbean	260 (5.2)	33.9 (21.8)	− 4.46	<0.001	− 7.18	− 1.73	− 1.57	0.08	− 3.34	0.20
South Asian	883 (17.6)	29.1 (21.3)	− 9.27	<0.001	− 10.81	− 7.72	− 2.64	<0.001	− 3.68	− 1.60
**Rheumatoid arthritis**										
White	7992 (91.7)	56.8 (15.4)	Ref.				Ref.			
Mixed-race/Other	148 (1.7)	47.6 (14.5)	− 9.13	<0.001	− 11.49	− 6.77	− 7.46	<0.001	− 9.63	− 5.29
African-Caribbean	159 (1.8)	49.4 (15.3)	− 7.39	<0.001	− 9.79	− 4.99	− 5.82	<0.001	− 8.14	− 3.50
South Asian	414 (4.8)	48.3 (14.8)	− 8.47	<0.001	− 9.94	− 7.01	− 7.14	<0.001	− 8.54	− 5.73
**Psoriasis**										
White	37,631 (92.8)	44.6 (19.0)	Ref.				Ref.			
Mixed-race/Other	820 (2.0)	37.9 (15.8)	− 6.64	<0.001	− 7.74	− 5.54	− 5.13	<0.001	− 6.09	− 4.18
African-Caribbean	405 (1.0)	37.3 (17.8)	− 7.31	<0.001	− 9.06	− 5.57	− 4.13	<0.001	− 5.43	− 2.82
South Asian	1685 (4.2)	38.2 (16.3)	− 6.41	<0.001	− 7.21	− 5.61	− 4.79	<0.001	− 5.43	− 4.15
**Pernicious anaemia**										
White	4382 (92.6)	61.5 (18.4)	Ref.				Ref.			
Mixed-race/Other	42 (0.9)	50.5 (18.0)	− 11.06	<0.001	− 16.47	− 5.65	− 8.43	<0.001	− 13.54	− 3.32
African-Caribbean	59 (1.3)	50.2 (16.6)	− 11.36	<0.001	− 15.61	− 7.12	− 10.51	<0.001	− 14.49	− 6.52
South Asian	248 (5.2)	53.1 (15.6)	− 8.40	<0.001	− 10.41	− 6.39	− 9.00	<0.001	− 10.97	− 7.02
**Myasthenia gravis**										
White	515 (92.5)	61.3 (16.3)	Ref.				Ref.			
Mixed-race/Other	8 (1.4)	30.0 (9.9)	− 31.37	<0.001	− 38.00	− 24.75	− 27.00	<0.001	− 31.91	− 22.08
African-Caribbean	13 (2.3)	38.8 (12.4)	− 22.53	<0.001	− 29.20	− 15.85	− 19.19	<0.001	− 25.08	− 13.31
South Asian	21 (3.8)	54.0 (20.4)	− 7.42	0.09	− 16.11	1.27	− 4.82	0.24	− 12.92	3.29
**Inflammatory bowel disease**										
White	7711 (92.6)	43.6 (18.3)	Ref.				Ref.			
Mixed-race/Other	137 (1.6)	33.6 (14.7)	− 9.95	<0.001	− 12.43	− 7.47	− 7.12	<0.001	− 9.26	− 4.98
African-Caribbean	104 (1.3)	37.7 (16.8)	− 5.90	<0.001	− 9.13	− 2.66	− 3.07	0.02	− 5.64	− 0.51
South Asian	380 (4.6)	36.2 (16.1)	− 7.41	<0.001	− 9.08	− 5.74	− 4.79	<0.001	− 6.15	− 3.43
**Coeliac**										
White	4994 (94.3)	40.9 (21.7)	Ref.				Ref.			
Mixed-race/Other	52 (1.0)	24.8 (15.4)	− 16.06	<0.001	− 20.26	− 11.86	− 9.49	<0.001	− 12.47	− 6.52
African-Caribbean	17 (0.3)	36.2 (20.2)	− 4.73	0.32	− 14.09	4.63	− 4.06	0.26	− 11.16	3.05
South Asian	234 (4.4)	32.7 (17.2)	− 8.21	<0.001	− 10.50	− 5.92	− 6.39	<0.001	− 8.00	− 4.78
**Autoimmune thyroid disease**										
White	3868 (87.0)	44.9 (14.7)	Ref.				Ref.			
Mixed-race/Other	223 (5.0)	41.8 (11.9)	− 3.05	<0.001	− 4.69	− 1.42	− 1.90	0.02	− 3.49	− 0.31
African-Caribbean	129 (2.9)	42.1 (11.8)	− 2.72	0.01	− 4.80	− 0.64	− 1.90	0.06	− 3.86	0.07
South Asian	226 (5.1)	38.7 (12.3)	− 6.13	<0.001	− 7.80	− 4.47	− 6.07	<0.001	− 7.63	− 4.51
**Multiple sclerosis**										
White	2374 (94.0)	44.0 (13.1)	Ref.				Ref.			
Mixed-race/Other	44 (1.7)	34.7 (10.6)	− 9.30	<0.001	− 12.44	− 6.16	− 8.40	<0.001	− 11.53	− 5.27
African-Caribbean	46 (1.8)	42.7 (12.4)	− 1.33	0.47	− 4.90	2.25	− 0.17	0.92	− 3.52	3.19
South Asian	62 (2.5)	37.1 (10.9)	− 6.92	<0.001	− 9.67	− 4.16	− 5.96	0.00	− 8.69	− 3.24
**Sjogren’s syndrome**										
White	902 (89.2)	56.9 (14.1)	Ref.				Ref.			
Mixed-race/Other	26 (2.6)	55.6 (12.5)	− 1.32	0.59	− 6.15	3.51	− 0.10	0.97	− 4.63	4.43
African-Caribbean	21 (2.1)	47.9 (10.7)	− 9.08	<0.001	− 13.64	− 4.51	− 8.21	<0.001	− 12.75	− 3.67
South Asian	62 (6.1)	44.8 (14.3)	− 12.16	<0.001	− 15.83	− 8.49	− 11.23	<0.001	− 14.77	− 7.70
**Fibromyalgia**										
White	10,603 (91.8)	45.7 (12.2)	Ref.				Ref.			
Mixed-race/Other	198 (1.7)	43.7 (10.9)	− 1.99	0.01	− 3.52	− 0.47	− 1.82	0.01	− 3.28	− 0.36
African-Caribbean	214 (1.9)	45.9 (11.5)	0.14	0.86	− 1.42	1.69	0.33	0.67	− 1.19	1.86
South Asian	531 (4.6)	44.2 (11.0)	− 1.54	<0.001	− 2.50	− 0.58	− 1.58	<0.001	− 2.53	− 0.64
**Autoimmune inflammatory diseases combined (ADs)**										
White	73,437 (90.9)	46.4 (19.3)	Ref.				Ref.			
Mixed-race/Other	1848 (2.3)	37.7 (16.7)	− 8.78	<0.001	− 9.55	− 8.01	− 6.12	<0.001	− 6.76	− 5.48
African-Caribbean	1275 (1.6)	39.7 (18.1)	− 6.74	<0.001	− 7.75	− 5.74	− 4.23	<0.001	− 5.00	− 3.45
South Asian	4216 (5.2)	37.7 (18.3)	− 8.71	<0.001	− 9.27	− 8.14	− 5.74	<0.001	− 5.30	− 6.18

^a^Adjusted for sex, deprivation, BMI categories and smoking status

### Rheumatic diseases

#### Rheumatoid arthritis

RA was diagnosed 7.1 years (95% CI=8.54 to −5.73, *p*=<0.001) earlier in the South Asian group than the White group, after adjustments for sex, BMI, smoking and deprivation (Table 2). RA was diagnosed 7.5 years (95% CI=5.29–9.63, *p*=<0.001) earlier in the Mixed-race/Other group relative to the White group, after statistical adjustments. The mean age at diagnosis was also significantly earlier by 5.8 years (95% CI= 3.5–8.14, *p*=<0.001) in the African-Caribbean group relative to the White group (Table 2).

#### Systemic lupus erythematosus and Sjogren’s syndrome

The total number of cases of SLE was low (*n*=95, *n*=99, *n*=78 in African-Caribbean, South Asian and Mixed-race/Other, respectively) in three of the ethnic groups compared to the White group (*n*=1213). Our data show that whilst the African-Caribbean group were diagnosed 4.7 years earlier (95% CI=1.76–7.69, *p*=<0.001), the South Asian and Mixed-race/Other groups had an even earlier age at diagnosis by 6.0 years (95% CI=3.06–8.93, *p*=<0.001) and 6.1 years (95% CI=3.28–8.87, *p*=<0.001), respectively (Table 2). Similarly, the number of cases of Sjogren’s syndrome was low in three of the ethnic groups (Table 2). We did not detect a significant difference in the diagnosis age of Sjogren’s between Mixed-race/Other and the White group, but the South Asian group were diagnosed 11.2 years (95% CI=7.70–14.77, *p*=<0.001) earlier.

### Gastrointestinal diseases

#### Inflammatory bowel disease and coeliac disease

There were significant differences in diagnosis ages of IBD between the different ethnic groups (Table 2). The age of IBD diagnosis was earlier, after adjustments, by 7.1 (95% CI=4.98–9.26, *p*=<0.001), 4.8 (95% CI=2.72–3.42, *p*=<0.001) and 3.1 (95% CI=0.15–5.13, *p*=<0.001) years in the Mixed-race/Other, South Asian and African-Caribbean groups, respectively. Our data indicated coeliac disease diagnosis age, after adjustments, to be significantly earlier by 6.4 years (95% CI=4.78–8.0, *p*=<0.001) in the South Asian group. The diagnosis age in the African-Caribbean group varied greatly as reflected by the large confidence interval and was not significantly different to the White group (*p* =0.26). In the Mixed-race/Other group, coeliac disease was diagnosed earlier by up to 9.5 years (95% CI=6.52–12.47, *P*=<0.001).

#### Pernicious anaemia

The age at diagnosis of PA was significantly earlier by 10.7 (95% CI=6.52–14.49, *P*=<0.001) years in the African-Caribbean group than the White group and earlier by 9 years (95% CI=7.02–10.97, *P*=<0.001) in the South Asian group compared with the White group. The diagnosis was earlier by 8.4 years (CI=3.32-13.54, *p*=<0.01) in the Mixed-race/Other group (Table 2).

### Neurological diseases

#### Multiple sclerosis and myasthenia gravis

There was no significant difference in MS diagnosis age between the African-Caribbean and White groups. Although the diagnosis was significantly earlier by 8.4 and 6.0 years in Mixed-race/Other and South Asian groups, respectively (Table 2). MG was diagnosed 27.0 years earlier in the Mixed-race/Other (*n*=8) group than the White group, although the number of cases was small. Similarly, a difference of 19.2 years was observed in the age at MG diagnosis in the African-Caribbean (*n*=13) group, but the age disparity was not as great (4.8 years) in the South Asian group (*n*=21).

### Immune-mediated skin conditions

#### Psoriasis

The diagnosis ages, after adjustments, were 5.1 (95% CI=4.18–6.09, *p*=<0.001), 4.8 (95% CI=4.15–5.43, *p*=<0.001) and 4.1 (95% CI=2.82–5.43, *p*=<0.001) years in Mixed-race/Other, South Asian and African-Caribbean groups, respectively, in comparison to the White group. The unadjusted diagnosis age showed a higher difference in diagnosis ages between the different ethnic groups (Table 3).

**Table 3:** Unadjusted and adjusted differences in age at disease diagnoses in different ethnic groups using White ethnicity as the reference group, data from UK Biobank

		Age/years	Unadjusted models				Adjusted models^a^			
	***N*** (%)	mean (SD)	Coefficient	***P***-val.	(95% CI)		Coefficient	***P***-val.	(95% Cl)	
**Systemic lupus erythematosus**										
White	888 (87.4)	49.8 (14.7)	Ref.				Ref.			
Mixed-race/Other	31 (3.1)	42.4 (12.6)	− 7.34	0.00	− 11.83	− 2.85	− 6.34	0.01	− 10.89	− 1.80
African-Caribbean	60 (5.9)	43.8 (13.8)	− 5.99	0.00	− 9.60	− 2.39	− 5.53	<0.01	− 9.23	− 1.84
South Asian	37 (3.6)	46.2 (14.5)	− 3.60	0.14	− 8.33	1.14	− 3.31	0.17	− 8.01	1.39
**Vitiligo**										
White	1016 (87.4)	48.7 (15.9)	Ref.				Ref.			
Mixed/other	35 (3.0)	50.2 (15.9)	1.52	0.57	− 3.78	6.83	0.80	0.76	− 4.37	5.97
African-Caribbean	24 (2.1)	51.3 (10.8)	2.63	0.24	− 1.72	6.98	1.96	0.39	− 2.53	6.44
South Asian	88 (7.6)	46.0 (14.5)	− 2.67	0.10	− 5.84	0.50	− 2.90	0.07	− 6.04	0.24
**Rheumatoid arthritis**										
White	11,173 (94.2)	55.7 (15.2)	Ref.				Ref.			
Mixed-race/Other	190 (1.6)	53.6 (14.9)	− 2.09	0.06	− 4.22	0.05	− 1.32	0.23	− 3.45	0.82
African-Caribbean	206 (1.7)	54.5 (13.8)	− 1.17	0.23	− 3.06	0.73	− 0.55	0.58	− 2.48	1.38
South Asian	296 (2.5)	54.1 (13.1)	− 1.56	0.04	− 3.07	− 0.04	− 1.31	0.09	− 2.84	0.21
**Psoriasis**										
White	14,466 (96.7)	45.1 (19.5)								
Mixed-race/Other	185 (1.2)	46.0 (15.7)	0.92	0.43	− 1.36	3.20	1.26	0.28	− 1.03	3.54
African-Caribbean	55 (0.4)	48.6 (15.1)	3.50	0.08	− 0.48	7.48	3.23	0.11	− 0.77	7.23
South Asian	260 (1.7)	48.6 (15.6)	3.49	0.00	1.56	5.41	3.32	<0.01	1.38	5.26
**Pernicious anaemia**										
White	3334 (93.6)	56.9 (12.7)	Ref.				Ref.			
Mixed-race/Other	24 (0.7)	52.0 (14.2)	− 4.81	0.09	− 10.40	0.78	− 3.73	0.15	− 8.86	1.41
African-Caribbean	28 (0.8)	54.7 (12.7)	− 2.14	0.37	− 6.79	2.51	− 2.10	0.32	− 6.23	2.02
South Asian	175 (4.9)	56.0 (11.5)	− 0.81	0.37	− 2.56	0.94	− 1.60	0.06	− 3.26	0.07
**Myasthenia gravis**										
White	465 (95.3)	57.3 (15.8)	Ref.				Ref.			
Mixed-race/Other	9 (1.8)	55.8 (6.0)	− 1.56	0.44	− 5.53	2.41	− 3.61	0.11	− 8.00	0.78
African-Caribbean	10 (2.0)	51.6 (10.2)	− 5.74	0.07	− 11.95	0.48	− 3.54	0.20	− 9.01	1.93
South Asian	4 (0.8)	62.8 (9.7)	5.41	0.21	− 3.03	13.86	5.93	0.32	− 5.81	17.67
**Inflammatory bowel disease**										
White	7252 (95.7)	48.9 (16.8)	Ref				Ref			
Mixed-race/Other	102 (1.4)	50.2 (16.0)	1.27	0.43	− 1.85	4.38	1.18	0.46	− 1.91	4.27
African-Caribbean	58 (0.8)	50.6 (13.4)	1.64	0.35	− 1.81	5.09	0.90	0.62	− 2.63	4.42
South Asian	163 (2.2)	49.2 (15.1)	0.26	0.83	− 2.08	2.61	− 0.03	0.98	− 2.39	2.34
**Coeliac**										
White	4609 (97.3)	56.3 (13.8)	Ref.				Ref.			
Mixed-race/Other	50 (1.1)	53.3 (11.2)	− 3.01	0.06	− 6.11	0.10	− 3.01	0.06	− 6.17	0.14
African-Caribbean	29 (0.6)	49.5 (15.5)	− 6.75	0.02	− 12.32	− 1.18	− 7.07	0.01	− 12.66	− 1.47
South Asian	51 (1.1)	52.2 (11.5)	− 4.03	0.01	− 7.18	− 0.88	− 4.31	0.01	− 7.45	− 1.16
**Multiple sclerosis**										
White	2410 (97.9)	44.7 (12.5)	Ref.				Ref.			
Mixed-race/Other	25 (1.0)	42.6 (9.5)	− 2.08	0.27	− 5.77	1.60	− 2.32	0.21	− 5.98	1.34
African-Caribbean	18 (0.7)	45.2 (9.1)	0.54	0.80	− 3.60	4.68	0.29	0.89	− 3.66	4.23
South Asian	10 (0.4)	51.3 (18.0)	6.62	0.22	− 3.97	17.20	7.00	0.19	− 3.55	17.55
**Sjogren’s syndrome**										
White	1015 (93.4)	64.4 (9.0)	Ref.				Ref.			
Mixed-race/Other	6 (0.6)	63.7 (7.4)	− 0.71	0.80	− 6.18	4.75	− 0.32	0.91	− 5.73	5.09
African-Caribbean	27 (2.3)	57.6 (10.1)	− 6.75	0.00	− 10.54	− 2.97	− 5.76	<0.01	− 9.54	− 1.99
South Asian	39 (3.6)	63.5 (9.3)	− 0.84	0.58	− 3.79	2.11	− 0.40	0.80	− 3.40	2.61
**Autoimmune inflammatory diseases combined (ADs)**										
White	42,664 (95.4)	50.0 (17.4)	Ref.				Ref.			
Mixed-race/Other	611 (1.4)	49.4 (15.2)	− 0.60	0.33	− 1.82	0.62	− 0.40	0.52	− 1.62	0.81
African-Caribbean	459 (1.0)	50.8 (14.2)	0.79	0.24	− 0.52	2.09	0.58	0.39	− 0.73	1.88
South Asian	1009 (2.3)	51.0 (14.5)	0.97	0.04	0.06	1.88	0.92	0.05	0.01	1.83

^a^Adjusted for sex, deprivation, BMI categories and smoking status

#### Vitiligo

The differences in the ages at diagnosis, without adjustments for covariates, were 9.3, 8.1 and 4.5 years earlier in the South Asian, Mixed-race/Other and African-Caribbean groups, respectively. In the adjusted models, the age disparity between the three ethnic groups was reduced to 2.6 (95% CI=1.60–3.68, *p*=<0.001), 2.3 (95% CI=0.80–3.88, *p*=<0.001) and 1.6 years (95% CI=0.20–3.34, *p*=0.08) earlier in South Asians, Mixed-race/Other and African-Caribbean groups, respectively, but was still statistically significant (Table 3).

### Autoimmune thyroid disease

There was no significant difference in the diagnosis age of AIT between the African-Caribbean and White groups. The diagnosis age difference was small, but significant, in Mixed-race/Other group (Table 3). Of the three non-White ethnic groups, the South Asian group differed the most in diagnosis age of AIT by 6.1 years (95% CI=4.51–7.63, *p*=<0.001).

### Non-IMD comparator disease

*Fibromyalgia:* We used FM as a comparator disease which is a common disorder of pain regulation but is not an autoimmune or inflammatory disease. The diagnosis of FM was slightly earlier by 1.6 (95% CI=0.64–2.54, *p*=<0.001) and 1.8 (95% CI=0.36–3.28, *p*=0.001) years in South Asian and Mixed-race/Other groups, respectively, in comparison to the White group (Table 2). However, there was no statistically significant difference in the ages at diagnosis between the African-Caribbean and White groups. Furthermore, removing cases of concomitant IMDs did not alter these results.

### Validation in UK Biobank

We used UKB to validate our observations and were able to confirm the earlier diagnosis in the three non-White ethnic groups for most IMDs although with a lower effect size (Table 3). However, we did not detect a difference in the diagnosis ages between the four ethnic groups when comparing all IMDs cases summed together (Table 3). Comparisons of diagnosis age could not be made for AIT and FM due to the lack of diagnosis dates in UKB. The IBD cases included ulcerative colitis and Crohn’s disease combined.

### Rheumatic diseases

#### Rheumatoid arthritis

RA was diagnosed earlier by 1.3, 0.6 and 1.3 years, after adjustments, in South Asian, African-Caribbean, and Mixed-race/Other groups, respectively, in comparison to the White group, although only reaching near significance for the South Asian group (Table 3).

#### SLE and Sjogren’s syndrome

The diagnosis ages of SLE were earlier by 5.5 (*p*=0.01) and 6.3 (*p*=<0.01) years in African-Caribbean and Mixed-race/Other groups, respectively. In contrast, SLE diagnosis age in the South Asian group, after adjustments, was 3.3 years earlier than the White group, though not significant (*p*=0.17). Interestingly for Sjogren’s syndrome, the diagnosis age was significantly earlier for the African-Caribbean group in comparison to the White group by 5.8 years (95% CI=1.99–9.54, *p*=<0.01). In contrast, there were no significant differences, in diagnosis ages, between the other three groups.

### Gastrointestinal diseases

#### Inflammatory bowel disease, coeliac disease

There were no significant differences in diagnosis ages of IBD between the four ethnic groups (Table 3). For Coeliac disease, the diagnosis age was earlier in all three non-White ethnic groups compared to the White group, which was statistically significant after adjustments for covariates (Table 3), with the African-Caribbean group diagnosed the earliest by 7.1 years (95% CI=1.47–12.66, *p*=0.01).

#### Pernicious anaemia

The diagnosis ages of PA were earlier in all three non-White groups in comparison to the White group although this difference was small and not significant (Table 3).

### Neurological diseases

#### Multiple sclerosis and myasthenia gravis

MS was diagnosed earlier by 2.3 years (95% CI= 1.34–5.98, *p*=0.21) in the Mixed-race/Other group but was 7.0 years later (95% CI=− 3.55–17.55, *p*=0.19), in the South Asian group than in the White group, though neither reached significance (Table 3). MG was diagnosed earlier by 3.5 and 3.6 years in the African-Caribbean and Mixed-race/Other groups, respectively, in comparison to the White group although not significant. The diagnosis age in the South Asian group was later by 5.9 years than in the White group, although again not significant (Table 3). The number of cases of MS and MG was small in all three ethnic groups making it difficult to draw conclusions (Table 3).

### Immune-mediated skin conditions

#### Psoriasis

Psoriasis was diagnosed later by 3.3 years (95% CI= 1.38–5.26, *p*=<0.01) in the South Asian group compared with the White group but the later diagnosis by 3.2 (95% CI=− 0.77–7.23, *p*=0.11) and 1.3 (95% CI=− 1.03–3.54, *p*=0.28) years in African Caribbean and Mixed-race/Other groups, respectively, were not significant.

#### Vitiligo

Vitiligo was diagnosed earlier by 2.9 years (95% CI= − 6.04–0.24, *p*=0.07) in South Asians compared to the White group; however, there were no statistical differences in the ages at diagnosis between the other ethnic groups.

## Discussion

Although it is known that the prevalence of IMDs varies by ethnicity, we did not find any publication that investigated the disparities in the ages at diagnoses between different ethnic groups. For example, an earlier age of onset of MS with higher disease severity scores was reported in the Black ethnic group in a US study, although the authors did not specify the degree of difference in the age of onset [18]. Two early studies of PA identified a much younger age of onset in Black and Latin American ethnic groups [19, 20] but without adjustments for confounding variables. This was also the case for SLE where the age at diagnosis was determined to be younger in the Black ethnic group (39.4 years, SD 15.9) in comparison to the White ethnic group (45.4 years, SD 17.7) but again with no adjustments for lifestyle factors [8].

Here we examined the ages at diagnosis of all common IMDs in four different ethnic groups in a UK cohort of ~4.5 million. We report for the first time that even after adjustment for potential lifestyle risk factors such as smoking, BMI and social deprivation, that South Asian, African-Caribbean, and Mixed-race/Other ethnicities have an earlier diagnosis of most IMDs in comparison to the White ethnic group. The number of years that diagnosis age is earlier by differs with the specific IMD and by ethnic group and ranges from 2 to 27 years.

We used UKB data to validate our findings and the trend was similar between the two data sets for most IMDs although the effect size was lower in the UKB cohort and for several IMDs did not reach significance. There are several possible explanations for this. Firstly, the number of participants in the UKB diagnosed with IMDs was lower (*n*=44,743) than in the IMRD-UK (*n*=80,776). Secondly, UKB is known to have a low representation of non-White ethnicities and is not representative of the UK population [21]. In UKB, the three non-White ethnic groups make up 4.7% (*n*=2079) of all IMDs cases whereas in the IMRD-UK dataset they represent 9% (*n*=7339) of all diagnosed IMDs. Thirdly, UKB participants are generally older and less likely to be from socioeconomically deprived areas [21]. Finally, the disease classification is slightly different in UKB from that in IMRD-UK. Read Codes were used to identify the specific IMDs within the IMRD-UK whilst ICD-10 codes were used to identify the different IMDs in UKB from either the hospital admissions records (HAR) or self-reports. Each of these factors could affect the determination of mean age at diagnosis.

However, there were also some inconsistencies between the IMRD-UK and UKB data. This includes psoriasis, which was actually diagnosed at a later age in the South Asian group in comparison to the White group and there were no differences in the ages at diagnosis between White, African-Caribbean and Mixed-race/Other ethnic groups. A potential explanation could be that of the 14,966 cases in UKB with diagnosis dates more than half were self-reports or from HAR records. It is possible that diagnosis dates obtained from HAR are secondary to another condition and therefore not reliable for determining the true age at diagnosis of psoriasis. Similarly, the self-reported diagnosis dates are less reliable as it is only the diagnosed condition that is verified by a healthcare professional and not the date. Removing cases with self-reported dates and diagnosis dates obtained from HAR reduced the number of cases to <10 in each of the three non-White ethnic groups, precluding statistical analysis. Similarly, for MG and MS, there was a disagreement between the two datasets for the South Asian group, but the number of cases was <10 in UKB making it difficult to draw conclusions.

A recent study from the US demonstrated that the incidence of IBD has increased by 39% and 134% in White and non-White individuals, respectively, between 1970 and 2010 [22]. Although we did not distinguish between the subtypes of IBD, we detected that the Mixed-race/Other group were diagnosed with IBD the earliest, by 7.1 years, in comparison to all other ethnic groups. This is also in agreement with a previous study which indicated that the median age at diagnosis for Crohn’s disease was the lowest in the Other ethnic group and highest in the White group [23] albeit not statistically significant possibly due to the small cohort size of the Other ethnic group (*n*=37).

A possible explanation for the observed earlier diagnosis age of IMDs in non-White groups, even after adjustments for BMI, could be the presence of a systemic pro-inflammatory state at an earlier age [24–27]. This hypothesis is supported by findings of only a slight difference in FM diagnosis age, a non-IMD, between the four ethnic groups. Previous large-cohort population studies have demonstrated that, irrespective of BMI, South Asians have higher levels of visceral, intermuscular and hepatic fat and significantly less total lean abdominal/back muscle mass than other ethnicities [28]. This could be a cause of the higher levels of pro-inflammatory adipokines such as resistin and lower levels of anti-inflammatory adiponectin detected in healthy individuals of this population [28, 29]. The combination of these factors likely contributes to a pro-inflammatory state as indicated by high hsCRP levels in South Asians [29]. However, exactly how increased inflammation could lead to earlier onset of IMDs is unclear. Inflammation has been shown to be one of the core processes driving ageing [30] and an intriguing possibility is that the biological ageing process occurs at different rates in different ethnicities. There are now methods to assess an individual’s biological as opposed to their chronological age based upon assessment of DNA methylation at specific sites, termed epigenetic clocks [31]. Such analyses have shown differences in biological ageing between ethnicities, for example Americans of Hispanic ethnicity had a higher extrinsic biological age compared to Whites and African Americans had a lower extrinsic biological age [32]. Ageing of the immune system, immunesenescence, is well documented and includes a predisposition to autoimmunity [33]. Immunesenescence both contributes to systemic inflammation and is increased by it and has been shown to occur at younger ages in IMDs such as RA [34]. The raised inflammation at an early age in non-White populations could accelerate immunesenescence and increase the risk of autoimmunity.

An additional explanation for earlier onset of IMDs in different ethnic minority groups includes the role of diet and the composition of the gut microbiome. Khine et al. compared the gut microbiome of pre-adolescents in Malaysia and China and reported a major influence of diet in the two ethnic groups [35], whilst other studies report that ethnicity per se influences the gut microbiome [36, 37]. The onset of IMD may be initiated subsequent to systemic inflammation which is highly influenced by the microbiome and is altered in several IMDs, including IBD and RA. RA patients have been shown to have a reduced diversity in the gut microbiota than healthy individuals [38]. Whilst certain bacterial species are more abundant in RA patients, others are scarce. For example, there is a higher composition of the bacterial species *Colinsella* sp., in RA patients and this has been shown to promote an increase in gut permeability and inflammation initially at a local level which then spreads to joints [38, 39]. Assessing microbiota diversity in different ethnic groups with IMDs would identify associations with disease prevalence and could suggest a role in the earlier age of onset.

Furthermore, other environmental factors such as psychosocial stress and socio-economic status may also contribute to the earlier onset of IMDs, though socio-economic status was taken into consideration in our analysis. This is supported by a recent Swedish population level sibling study that detected an association between stress-related disorders and an increased risk of IMDs [40]. Similar associations between post-traumatic stress disorders and an increased risk of IMDs have also been reported in large cohort studies [40, 41]. Lastly, the latest mendelian randomisation studies have reported a higher educational attainment with a protective benefit towards risk of RA [42]. The level of educational attainment is known to be affected by socio-economic inequalities. However, further studies are required to fully assess the risk of IMDs attributed to individual socio-economic factors and the earlier onset of disease.

Finally, a further potential explanation could be that patients from ethnic minority groups may have a different mode of onset of disease (e.g., a more abrupt and severe onset, as opposed to an insidious and mild onset). It is also likely that the measures of the ‘mode of disease onset’, ‘disease severity’ or ‘disease activity’ will be different across the different IMDs and future studies should use approaches to capture these constructs that are specific for each of the IMDs.

### Limitations of study

The study limitations include missing ethnicity data however, this should not compromise the observations in this study as we assume the missing data to be absent from all ethnic groups and not disproportionately from a specific ethnic group. Furthermore, the proportional representation of the non-White groups reflects the UK population as per 2011 census data. Although, self-reported ethnicity could be a potential confounder.

Some IMDs such as SLE and Sjogren’s syndrome take several years to be diagnosed due to initial non-specific symptoms [43]. It is therefore likely that the onset of disease is substantially earlier than the age at diagnosis. An additional issue, particularly in the South Asian group, is patient’s reluctance to seek medical help early, which inevitably means a later diagnosis after disease onset [44–47]. Together these factors imply that the actual age of onset of IMDs is likely to be even earlier, by perhaps several years, than the diagnosis age in certain ethnic groups.

Another limitation is that our ethnicity grouping is broad, and it is likely that there is variation in diagnosis ages within each ethnic grouping. This is especially true for the Mixed-race/Other group, which includes several diverse ethnic groups and potentially dilutes the overall effect.

Using Read Codes for case definitions may also be imperfect and there may be a small amount of misclassification of IMDs, for example by including some patients with a non-autoimmune aetiology. To mitigate this as much as possible we used Read Codes which had been validated previously [3, 16]. Lastly, the number of cases of the rarer IMDs such as SLE, MG and Sjogren’s syndrome were very small, although the earlier age at SLE diagnosis was detected in all three ethnic groups in comparison to the White group, consistent with previous studies [48].

## Conclusions

In conclusion, we observed an earlier age at diagnosis for almost all IMDs in non-White ethnic groups in comparison to the White group. Earlier onset of disease may suggest differing pathogenesis with ethnicity. The healthcare implication would be to screen patients from non-White ethnic groups at an earlier age to allow novel treatment interventions such as reversal of the ageing processes if accelerated biological ageing was identified as the cause of the earlier onset of IMDs. Earlier intervention may delay disease progression to severe disease and disability due to IMDs.

## Supplementary Information

**Additional file 1. **A list of the Read codes used to identify all cases of each of the IMDs used in the study from IMRD.

**Additional file 2: Table S1.** The ICD-10 codes used to identify all cases of each of the IMDs, used in the study, from UKB.

## Data Availability

Presented within the manuscript and as supplementary table and Additional file.

## Abbreviations

IMDs: Immune-mediated diseases (autoimmune inflammatory diseases)
AIT: Autoimmune thyroid disease
BMI: Body mass index
EHR: Electronic health records
FM: Fibromyalgia
HAR: Hospital admissions records
IBD: Inflammatory bowel disease
IMRD-UK: IQVIA Medical Research Data
IQR: Interquartile range
MG: Myasthenia gravis
MS: Multiple sclerosis
PA: Pernicious anaemia
RA: Rheumatoid arthritis
SLE: Systemic lupus erythematosus
