# Supplementary material for: Early onset of immune-mediated diseases in minority ethnic groups in the UK
Source: BMC Med. 2022 Oct 13;20:346. doi: 10.1186/s12916-022-02544-5 (PMC9558944; doi:10.1186/s12916-022-02544-5)
Supplement: Supplementary file 1 — Additional file 1. A list of the Read codes used to identify all cases of each of the IMDs used in the study from IMRD. [file 12916_2022_2544_MOESM1_ESM.docx]

**Read code lists used to define AIDs and fibromyalgia**

1. OA - Omitted

"2.) Fibromyalgia" : {

"codes" : "{N239.00=Fibromyalgia, N239.11=Myofascial pain syndrome, N248000=Myofascial pain syndrome, N248.00=Fibromyalgia}"

},

"3.) SLE" : {

"codes" : "{=, N000.00=Systemic lupus erythematosus, N000100=Libman-Sacks disease, N000000=Disseminated lupus erythematosus, M154.00=Lupus erythematosus, K01x400=Nephrotic syndrome in systemic lupus erythematosus, M154000=Lupus erythematosus chronicus, M154100=Discoid lupus erythematosus, Myu7800=[X]Other local lupus erythematosus, K0B4000=Renal tubulo-interstitial disorder in SLE, M154200=Lupus erythematosus migrans, F371000=Polyneuropathy in disseminated lupus erythematosus, F396100=Myopathy due to disseminated lupus erythematosus, M154300=Lupus erythematosus nodularis, M154400=Lupus erythematosus profundus, M154500=Lupus erythematosus tumidus, N000z00=Systemic lupus erythematosus NOS, M154600=Lupus erythematosus unguium mutilans, M154700=Subacute cutaneous lupus erythematosus, M154z00=Lupus erythematosus NOS, N000300=Systemic lupus erythematosus with organ or sys involv, N000500=Neonatal lupus erythematosus, N000400=Systemic lupus erythematosus with pericarditis, N000600=Cerebral lupus, K01x411=Lupus nephritis, F4D3300=Eyelid discoid lupus erythematosus, H57y400=Lung disease with systemic lupus erythematosus, N000611=Systemic lupus erythematosus encephalitis}"

},

"4.) Sjogrens" : {

"codes" : "{N002.00=Sicca (Sjogren's) syndrome, H57y300=Lung disease with Sjogren's disease, F396700=Myopathy due to Sjogren's disease}"

},

"5.) CPRD_vitiligo" : {

"codes" : "{F4E5311=Vitiligo of eyelid, M295100=Vitiligo}"

},

"6.) RheumatoidArthritis" : {

"codes" : "{N040100=Other rheumatoid arthritis of spine, N040.00=Rheumatoid arthritis, N040000=Rheumatoid arthritis of cervical spine, N040400=Rheumatoid arthritis of acromioclavicular joint, N040500=Rheumatoid arthritis of elbow, N040200=Rheumatoid arthritis of shoulder, N040300=Rheumatoid arthritis of sternoclavicular joint, N040800=Rheumatoid arthritis of MCP joint, N040900=Rheumatoid arthritis of PIP joint of finger, N040600=Rheumatoid arthritis of distal radio-ulnar joint, N040700=Rheumatoid arthritis of wrist, N040A00=Rheumatoid arthritis of DIP joint of finger, N040D00=Rheumatoid arthritis of knee, N040E00=Rheumatoid arthritis of tibio-fibular joint, N040B00=Rheumatoid arthritis of hip, N040C00=Rheumatoid arthritis of sacro-iliac joint, N040H00=Rheumatoid arthritis of talonavicular joint, N040F00=Rheumatoid arthritis of ankle, N040G00=Rheumatoid arthritis of subtalar joint, N040L00=Rheumatoid arthritis of lesser MTP joint, N040M00=Rheumatoid arthritis of IP joint of toe, N040J00=Rheumatoid arthritis of other tarsal joint, N040K00=Rheumatoid arthritis of 1st MTP joint, N040P00=Seronegative rheumatoid arthritis, N040Q00=Rheumatoid bursitis, N040N00=Rheumatoid vasculitis, N040T00=Flare of rheumatoid arthritis, N040R00=Rheumatoid nodule, N040S00=Rheumatoid arthritis - multiple joint}"

},

"7.) CPRD_psoriasis" : {

"codes" : "{14F2.00=H/O: psoriasis, M16y000=Scalp psoriasis, Myu3000=[X]Other psoriasis, M161H00=Erythrodermic psoriasis, M166.00=Palmoplantar pustular psoriasis, M161J00=Flexural psoriasis, M161D00=Pustular psoriasis, M161C00=Psoriasis punctata, M16y.00=Other psoriasis and similar disorders, M161F00=Psoriasis vulgaris, M161E00=Psoriasis universalis, M161B00=Psoriasis plantaris, M161A00=Psoriasis palmaris, M160z00=Psoriatic arthropathy NOS, M160200=Arthritis mutilans, M16..00=Psoriasis and similar disorders, M160.00=Psoriatic arthropathy, M160000=Psoriasis spondylitica, M160100=Distal interphalangeal psoriatic arthropathy, M161.00=Other psoriasis, Nyu1300=[X]Other psoriatic arthropathies, M16z.00=Psoriasis and similar disorders NOS, M161F11=Chronic large plaque psoriasis, N045200=Juvenile arthritis in psoriasis, M161800=Psoriasis inveterata, M161700=Psoriasis gyrata, M161z00=Psoriasis NOS, M161900=Psoriasis ostracea, M161400=Psoriasis discoidea, M161300=Psoriasis diffusa, M161600=Guttate psoriasis, M161500=Psoriasis geographica, M161000=Psoriasis unspecified, M160.11=Psoriatic arthritis, M161200=Psoriasis circinata, M161100=Psoriasis annularis}"

},

"8.) PerniciousAnaemia" : {

"codes" : "{=, F381500=Myasthenic syndrome due to pernicious anaemia, D010.00=Pernicious anaemia, D010.11=Addison's anaemia}"

},

"9.) CPRD_myasthenia" : {

"codes" : "{F380100=Juvenile or adult myasthenia gravis, F380z00=Myasthenia gravis NOS, F380.00=Myasthenia gravis}"

},

"10.) InflammatoryBowelDisease" : {

"codes" : "{J400.00=Regional enteritis of the small bowel, J410z00=Ulcerative proctocolitis NOS, J400000=Regional enteritis of the duodenum, J400100=Regional enteritis of the jejunum, J40..00=Regional enteritis - Crohn's disease, J402.00=Regional ileocolitis, J41z.00=Idiopathic proctocolitis NOS, J401z11=Crohn's colitis, J400200=Crohn's disease of the terminal ileum, J400300=Crohn's disease of the ileum unspecified, J400400=Crohn's disease of the ileum NOS, J400500=Exacerbation of Crohn's disease of small intestine, J412.00=Ulcerative (chronic) ileocolitis, J08z900=Orofacial Crohn's disease, J400z00=Crohn's disease of the small bowel NOS, J410000=Ulcerative ileocolitis, J41..00=Idiopathic proctocolitis, J410.00=Ulcerative proctocolitis, J410300=Ulcerative proctitis, J410400=Exacerbation of ulcerative colitis, J410100=Ulcerative colitis, J410200=Ulcerative rectosigmoiditis, J41yz00=Other idiopathic proctocolitis NOS, J40z.00=Regional enteritis NOS, J401000=Regional enteritis of the colon, J401100=Regional enteritis of the rectum, J401200=Exacerbation of Crohn's disease of large intestine, J40..12=Granulomatous enteritis, J40..11=Crohn's disease, J401.00=Regional enteritis of the large bowel, J401z00=Crohn's disease of the large bowel NOS, J41y.00=Other idiopathic proctocolitis, J413.00=Ulcerative pancolitis, Jyu4000=[X]Other Crohn's disease, Jyu4100=[X]Other ulcerative colitis, J41..11=Mucous colitis and/or proctitis, J411.00=Ulcerative (chronic) enterocolitis, J41..12=Ulcerative colitis and/or proctitis, J40z.11=Crohn's disease NOS}"

},

"11.) AutoimmuneThyroid" : {

"codes" : "{Cyu1400=[X]Other chronic thyroiditis, C05..00=Thyroiditis, C020.11=Basedow's disease, C050000=Acute nonsuppurative thyroiditis, C020000=Toxic diffuse goitre with no crisis, C020.12=Graves' disease, C020200=Thyroid-associated dermopathy, C020100=Toxic diffuse goitre with crisis, C052.11=Autoimmune thyroiditis, C052.00=Chronic lymphocytic thyroiditis, C052.12=Hashimoto's disease, C020z00=Toxic diffuse goitre NOS, C050.00=Acute thyroiditis, C051.11=De Quervain's thyroiditis, C051.00=Subacute thyroiditis, C053.00=Chronic fibrous thyroiditis, C053.11=Riedel's thyroiditis, C05y400=Chronic thyroiditis with transient thyrotoxicosis, C05z.00=Thyroiditis NOS, C05y.00=Other and unspecified chronic thyroiditis, C050z00=Acute thyroiditis NOS, C046.00=Autoimmune myxoedema}"

},

"12.) MultipleSclerosis" : {

"codes" : "{F201.00=Multiple sclerosis of the spinal cord, F202.00=Generalised multiple sclerosis, F200.00=Multiple sclerosis of the brain stem, F20..00=Multiple sclerosis, 666A.00=Multiple sclerosis review, 666B.00=Multiple sclerosis multidisciplinary review, F206.00=Primary progressive multiple sclerosis, F20z.00=Multiple sclerosis NOS, F203.00=Exacerbation of multiple sclerosis, F204.00=Benign multiple sclerosis, 8CS1.00=Multiple sclerosis care plan agreed, F207.00=Relapsing and remitting multiple sclerosis, F208.00=Secondary progressive multiple sclerosis}"

},

"13.) CPRD_coeliac" : {

"codes" : "{J690.12=Gee - Herter disease, J690000=Congenital coeliac disease, J690.13=Gluten enteropathy, J690100=Acquired coeliac disease, J690.14=Sprue - nontropical, J690.15=Steatorrhea - idiopathic, J690.00=Coeliac disease, 6648.00=Coeliac disease monitoring, J690.11=Coeliac rickets, 6648000.0=, 9mB..00=Coeliac disease monitoring invitation, 9mB1.00=Coeliac disease monitoring invitation first letter, 8IAp.00=Coeliac disease annual review declined, J690z00=Coeliac disease NOS, ZC2C200=Dietary advice for coeliac disease}"

}

},
