# Supplementary material for: Early onset of immune-mediated diseases in minority ethnic groups in the UK
Source: BMC Med. 2022 Oct 13;20:346. doi: 10.1186/s12916-022-02544-5 (PMC9558944; doi:10.1186/s12916-022-02544-5)
Supplement: Supplementary file 2 — Additional file 2: Table S1. The ICD-10 codes used to identify all cases of each of the IMDs, used in the study, from UKB. [file 12916_2022_2544_MOESM2_ESM.docx]

**Table 1.** ICD-10 codes used to identify the common AIDs in UKB.

| **Biobank data field** | **ICD-10 code** | **Disease Type** |
| --- | --- | --- |
| 131894 | M32 | Systemic lupus erythematosus |
| 131802 | L80 | Vitiligo |
| 131848 and 131850 | M05 and M06 | Rheumatoid arthritis |
| 130624 | D51 | Pernicious Anaemia |
| 131742 | L40 | Psoriasis |
| 131626 and 131628 | K50 and K51 | Inflammatory bowel disease |
| 131092 | G70 | Myasthenia Gravis |
| 131688 | K90 | Coeliac |
| 130702 | E06 | Thyroiditis |
| 131042 | G35 | Multiple sclerosis |
| 131900 | M35.0 | Sjogren’s syndrome |
